# Heterologous protein display on the cell surface of lactic acid bacteria mediated by the s-layer protein

**DOI:** 10.1186/1475-2859-10-86

**Published:** 2011-10-28

**Authors:** Shumin Hu, Jian Kong, Zhilan Sun, Lanlan Han, Wentao Kong, Pu Yang

**Affiliations:** 1 State Key Laboratory of Microbial Technology, Shandong University, Jinan, P. R. China; 2 Scientific Research Center, Tsingtao Brewery Co. Ltd, Qingdao, P. R. China

## Abstract

**Background:**

Previous studies have revealed that the C-terminal region of the S-layer protein from *Lactobacillus *is responsible for the cell wall anchoring, which provide an approach for targeting heterologous proteins to the cell wall of lactic acid bacteria (LAB). In this study, we developed a new surface display system in lactic acid bacteria with the C-terminal region of S-layer protein SlpB of *Lactobacillus crispatus *K2-4-3 isolated from chicken intestine.

**Results:**

Multiple sequence alignment revealed that the C-terminal region (LcsB) of *Lb. crispatus *K2-4-3 SlpB had a high similarity with the cell wall binding domains S_A _and CbsA of *Lactobacillus acidophilus *and *Lb. crispatus*. To evaluate the potential application as an anchoring protein, the green fluorescent protein (GFP) or beta-galactosidase (Gal) was fused to the N-terminus of the LcsB region, and the fused proteins were successfully produced in *Escherichia coli*, respectively. After mixing them with the non-genetically modified lactic acid bacteria cells, the fused GFP-LcsB and Gal-LcsB were functionally associated with the cell surface of various lactic acid bacteria tested. In addition, the binding capacity could be improved by SDS pretreatment. Moreover, both of the fused proteins could simultaneously bind to the surface of a single cell. Furthermore, when the fused DNA fragment of *gfp:lcsB *was inserted into the *Lactococcus lactis *expression vector pSec:Leiss:Nuc, the GFP could not be secreted into the medium under the control of the *nisA *promoter. Western blot, in-gel fluorescence assay, immunofluorescence microscopy and SDS sensitivity analysis confirmed that the GFP was successfully expressed onto the cell surface of *L. lactis *with the aid of the LcsB anchor.

**Conclusion:**

The LcsB region can be used as a functional scaffold to target the heterologous proteins to the cell surfaces of lactic acid bacteria *in vitro *and *in vivo*, and has also the potential for biotechnological application.

## Background

Surface layers (S-layers), which are composed of protein or glycoprotein subunits, are the outmost cell surface structure of many Bacteria and Archaea. They can self-assemble spontaneously into the crystalline lattices in solutions, on various solid surfaces, and interfaces [1]. Several species of the genus *Lactobacillus *possess an S-layer protein with molecular masses ranging from 25 to 71 kDa, the smallest known proteins considered to function in the adherence to the intestinal epithelial cells and mammalian extracellular matrix [2].

S-layer proteins normally contain two functional regions: the self-assembly domain and the cell wall-targeting domain. These regions have been characterized in the S-layer proteins S_A _of *Lb. acidophilus *ATCC 4356 [3], CbsA of *Lb. crispatus *JCM 5810 [4], and SlpA of *Lactobacillus brevis *ATCC 8287 [5]. The sequences of S_A _and CbsA showed identity especially in the C-terminal regions, which were proved to anchor the S-layer subunits to the bacterial cell wall, and the more variable N-terminal regions were involved in the self-assembly process [3,4]. In the S-layer proteins of lactobacilli, no S-layer homology (SLH) motif at the C-terminus, which was responsible for the non-covalent attachment of the protein subunits to the cell wall through a pyruvylated polysaccharide receptor in the cell wall [6], has been identified. Instead, an interaction between basic amino acids in the cell wall binding region and negatively charged secondary cell wall polymers has been proposed to mediate the cell wall binding in the case of S_A _and CbsA. The (lipo)teichoic acids have been proved to be the cell wall ligands of S_A _and CbsA [3,4]. In contrast, in SlpA of *Lb. brevis *ATCC 8287, the domains responsible for the self-assembly process (C-terminal region) and for cell wall binding (N-terminal region) were located in reverse order compared to S_A _and CbsA. The specific cell wall component that interacts with the S-layer proteins of *Lb. brevis *ATCC 8287 and *Lactobacillus buchneri *was shown to be the neutral polysaccharide moiety of the cell wall [5,7].

The display of heterologous proteins on the cell surface of lactic acid bacteria (LAB) is an exciting and emerging research area that holds great promise for a variety of biotechnological applications, including the developments of live vaccine delivery system, diagnostics, peptide library screening and whole-cell biocatalysts [8]. Various anchoring proteins have also been explored for their efficiency in attaching hybrid proteins to the cell membrane or cell wall of LAB, such as LPXTG-containing proteins [9], transmembrane protein PgsA [10], LysM motif-containing proteins [11], S-layer proteins [12]. The LPXTG-containing proteins have been the most commonly used anchor in most of the previously reported cell surface display systems. The functional proteins fused to the cell wall anchor were initially synthesized and anchored to the cell surface of producing hosts after their translocation across the membrane. However, the expressed proteins were anchored to the producer cells, thus making the host strain for surface display a genetically modified organism (GMO) [9,13]. Other studies have provided insight into the use of the non-genetically modified gram-positive bacterial cells as matrixes to bind externally added heterologous proteins by means of a high-affinity binding domain. These novel tools were based on the peptidoglycan-binding domain of the major autolysin AcmA from *Lactococcus lactis *and the endolysin Lyb5 of *Lactobacillus fermentum *bacteriophage ΦYB5 which contained three LysM repeats in theirs C terminus [14-16]. The cell wall anchor fused to the foreign proteins was overproduced in a model organism (e.g., *E. coli*), and interestingly found to maintain its capacity to anchor the functional proteins to the cell surface of LAB. These novel surface display systems opened the possibility of surface display of foreign proteins on the non-genetically modified LAB cells.

S-layer proteins as an anchor device for surface display of functional proteins have been reported previously [12,17-19]. Most of the applications were based on the fusions between the desired functional molecules with S-layer proteins. The S-layer proteins were not affected by the fusion partner molecules, while the functional sequences were represented on the outermost surface of the S-layer lattice. The truncated forms of the S-layer protein SbsA and SbsB from *Bacillus stearothermophilus *were capable of carrying amino acid insertion of up to 500 residues without any effect on their self-assembly capacity when the fusion proteins were produced in *E. coli *[20]. *Lactobacillus *S-layer epitope constructs have been expressed in the original hosts. The small epitopes VP1, a poliovirus epitope, and c-Myc, which comprised 10 and 11 amino acid residues, respectively, were successfully displayed on the cell surface as part of the S-layer proteins of lactobacilli. Although a heterologous epitope was present in every S-layer subunit, an intact S-layer could be formed without any change in the crystalline structure [12].

In this study, we developed an approach for surface display of the heterologous proteins on the LAB cells by means of the C-terminal region of S-layer protein SlpB (LcsB) from *Lb. crispatus *K2-4-3. The LcsB fusion protein (GFP-LcsB) was produced in *E. coli *and was able to bind to the cell walls of LAB *in vitro*, demonstrating that the LcsB has the ability of binding the heterologous protein to the cell surface. The system was then further applied to display of beta-galactosidase with enzymatic properties, as well as those two proteins (GFP and Gal) simultaneously. Furthermore, we also evaluated the function of LcsB by constructing the cell wall-targeting expression vector pLeiss:GFP:LcsB, which could express the fusion GFP-LcsB under the control of *nis*A promoter in *L. lactis*. Western blot, in-gel fluorescence assay, immunofluorescence microscopy and SDS sensitivity analysis showed that the GFP was exposed on the cell surface of *L. lactis*.

## Results

### The primary analysis of the S-layer protein SlpB from *Lb. crispatus *isolated from chicken intestine

The presence of a putative S-layer protein on the bacterial cell surface can be deduced from the occurrence of a dominant protein band in the sodium dodecyl sulfate (SDS)-extractable protein profile of non-lysed bacteria. Dominant protein bands of 45-62 kDa were present in 13 out of the 45 isolates, suggesting the presence of S-layer proteins in these strains (data not shown). The presence of the S-layer protein in the strain *Lb. crispatus *K2-4-3 with high-adhesive capacity was demonstrated by polymerase chain reaction (PCR) targeting the S-layer protein gene. The whole S-layer protein gene (*slpB*) of *Lb. crispatus *K2-4-3 was obtained by ligation-anchored PCR from the genomic DNA. The *slpB *was found to consist of 1332 nucleotides and to have the capacity to encode a polypeptide of 440 amino acid residues, which was identified and showed 95% identity with the silent S-layer protein of *Lb. crispatus *JCM 5810 [21]. A signal sequence of 57 amino acid residues was identified on the deduced amio acid sequence. According to the 16S rRNA gene sequence analysis, *Lb. crispatus *K2-4-3 was identified as *Lb. crispatus*. The GenBank accession numbers of the sequences obtained in this study were HQ716719 and HQ716720.

**Figure 1:**
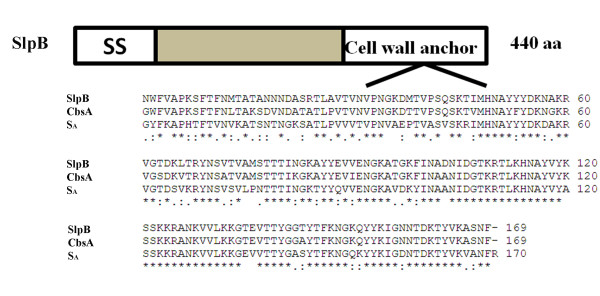
**Schematic representation of the SlpB protein and sequence alignment of different S-layer proteins**. In the schematic drawing of the S-layer protein SlpB comprising 440 amino acids, the signal sequence (SS) as well as the cell wall anchor are indicated. As demonstrated by multiple sequence alignment, the sequences of the C-terminally located cell wall anchor regions from the S-layer proteins SlpB of *Lb. crispatus *K2-4-3, S_A _of *Lb. acidophilus *ATCC 4356 and CbsA of *Lb. crispatus *JCM 5810 showed high identity.

Previous studies have also shown that the conserved regions in the C-terminus of S-layer proteins of *Lb. acidophilus, Lb. crispatus *and *Lactobacillus helveticus *were involved in the cell wall binding [3,4]. According to the multiple sequence alignment in Figure 1, the C-terminal region of SlpB of *Lb. crispatus *K2-4-3 (LcsB) obtained in this study had a high degree of similarity with the conserved C-terminal domain S_A _of *Lactobacillus acidophilus *and CbsA *Lb. crispatus*, suggesting that the LcsB region has the potential in anchoring the foreign protein to the cell wall.

### GFP displaying on the cell surface of various LAB mediated by the LcsB region

**Figure 2:**
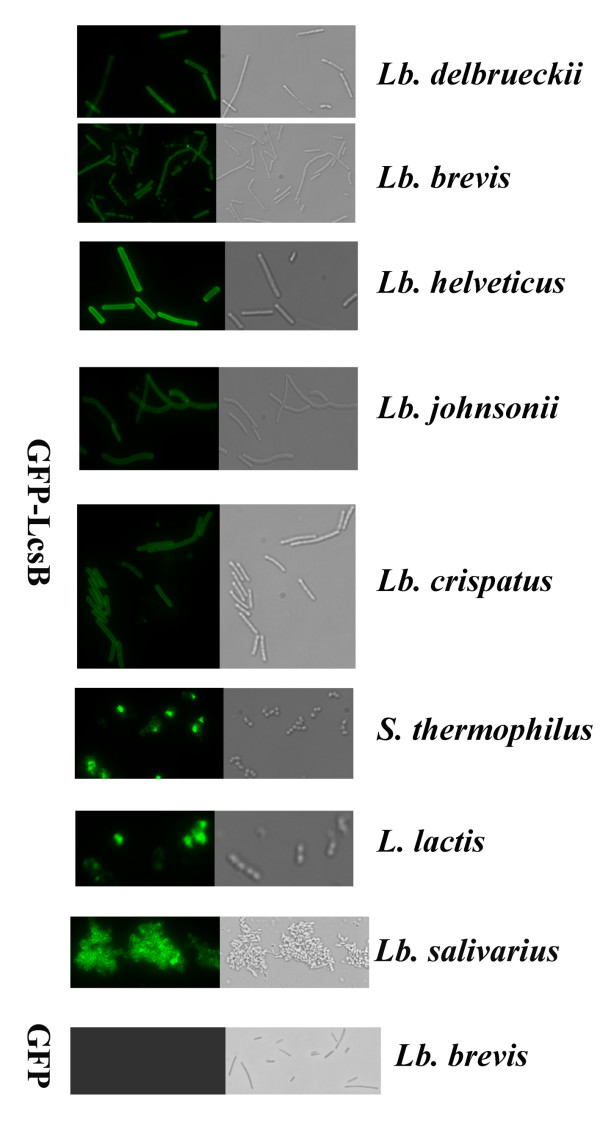
**GFP-LcsB binds to the surface of various lactic acid bacteria cells**. The GFP-LcsB proteins were added to various LAB cells (*Lb. delbrueckii*, *Lb. brevis*, *Lb. helveticus*, *Lb. johnsonii*, *Lb. crispatus*, *S. thermophilus*, *L. lactis *and *Lb. salivarius*) and mixed for 1 hour. Then, the cells were collected, washed and resuspended in PBS. Subsequently, the cells were observed by fluorescence microscopy.

To evaluate the possibility of the LcsB region as a cell wall anchor, the LcsB region was tagged with green fluorescent protein (GFP), a convenient tool to investigate the protein localization in cells [22]. The GFP (27 kDa) and GFP-LcsB (47 kDa) were firstly overproduced in *E. coli *(data not shown). Subsequently, the purified GFP or GFP-LcsB was added to various lactic acid bacteria cells *in vitro*, and the binding was viewed by fluorescence microscopy. Surprisingly, GFP-LcsB bound to the cells of *Lactobacillus delbrueckii*, *Lb. brevis*, *Lb. helveticus*, *Lactobacillus johnsonii*, *Lb. crispatus*, *Streptococcus thermophilus*, *L. lactis *and *Lactobacillus salivarius*, causing fluorescence of cell surfaces. As a negative control, GFP alone did not bind to cells. These results indicated that the GFP could be bound to the cell surface of LAB tested under the direction of the LcsB region (Figure 2). Nevertheless, the fluorescence of the *Lactobacillus *cells was more intensive than that of the *L. lactis *and *S. thermophilus*, especially those of the S-layer-containing strains (*Lb. brevis*, *Lb. helveticus*, *Lb. johnsonii *and *Lb. crispatus*). In addition, in the case *Lb. salivarius*, the cells formed aggregates after mixing with the GFP-LcsB fusion protein. Moreover, GFP-LcsB was not displayed on the cell surface of *Lb. casei*, which was consistent with the previous report that the CbsA protein of *Lb. crispatus *was not retained at the cell surface of *Lb. casei *and secreted into the medium [23]. Comparing with the LysM motif-containing anchor [14,15], the GFP-LcsB was located equally at the whole surface of cells, suggesting the cell wall ligand was different between the LcsB and Ly5C anchor.

### Investigation of the LcsB-binding capacity by LAB cells after chemical pretreatment

**Figure 3:**
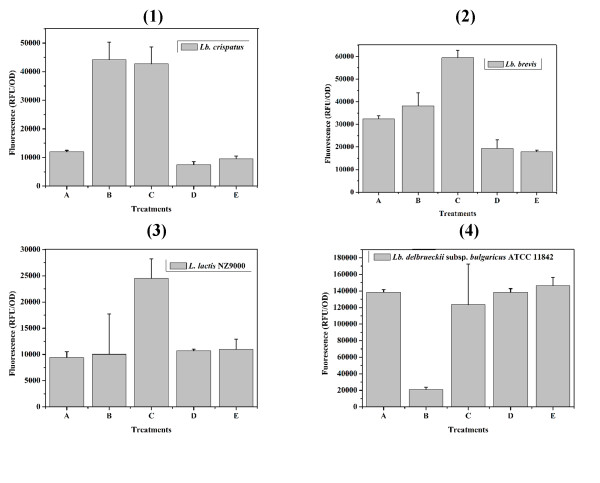
**Effects of different chemical pretreatments on the LcsB-binding capacity of LAB strains**. The LAB strains *Lb. crispatus *(1), *Lb. brevis *(2), *L. lactis *(3), and *Lb. delbrueckii *(4) were investigated for their LcsB binding capacity after chemical treatment of the cells. Bar A, heating at 100°C for 10 min; bar B, 5% TCA at 100°C for 10 min; bar C, 10% SDS at 100°C for 10 min; bar D, no treatment; bar E, 90% acetone at room temperature for 10 min. The data points represent the averages and standard deviations from four independent experiments.

Trichloroacetic acid (TCA) is known to remove mainly (lipo)teichoic acids from the cell wall and SDS has the ability of removing the cell-wall associated proteins (e.g. the S-layer protein) [14,15]. To enhance the binding capacity of LcsB and simply analyze the cell wall receptors of LcsB, various LAB cells were pretreated with SDS, TCA and acetone. Compared to nonpretreated cells of *Lb. cripatus*, *Lb. brevis *and *L. lactis*, 2-fold increases in fluorescence intensity were obtained with SDS pretreated cells, illustrating that SDS pretreatment was efficient in enhacing the LcsB binding capacity (Figure 3). Moreover, the effect of TCA pretreatment was different in the loading capacity of the LcsB anchor for different strains tested. For *Lb. delbrueckii*, TCA pretreatment showed a great decrease in fluorescence intensity, indicating that the lipo(teichoic) acid rather than lipid or cell-associtaed proteins was involved in the binding process. However, the increase in fluorescence intensity was obtained from the cells of *Lb. crispatus *pretreated by TCA, and no change in the fluorescence intensity of *L. lactis *cells was observed no matter whether the cells were pretreated or not.

**Figure 4:**
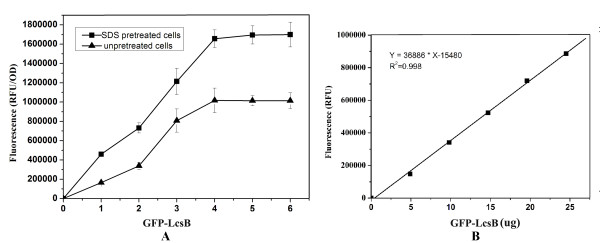
**Quantificational detection of surface-displayed GFP-LcsB fusion proteins**. (A) Different amounts of purified GFP-LcsB were added to an equal amount of SDS-pretreated and nonpretreated *Lb. brevis *cells for 1 hour, respectively. Subsequently, the cells were collected, washed and resuspended in 1 mL PBS. 100 μL samples were added to the test plate to measure the fluorescence and cell density at 600 nm, respectively. (B) Standard curve of the fluorescence and the protein concentration of GFP-LcsB fusion protein. The data points represent the averages and standard deviations from four independent experiments.

To determine the maximum binding capacity, various amounts of purified GFP-LcsB fusion proteins were mixed with the SDS pretreated and nonpretreated cells of *Lb. brevis *with an optical density at 600 nm (OD_600_) of 1 (approximate 10^8 ^cfu/mL). As shown in Figure 4, the cells of SDS pretreated and nonpretreated *Lb. brevis *could bind approximately 475 μg and 275 μg of GFP-LcsB fusion proteins, respectively. Therefore, each cell could bind approximately 10^7 ^GFP-LcsB molecules, which had a 10-fold increment by comparison with the Ly5C mediated surface display system [16].

### Displaying beta-galactosidase on the cell surface of LAB

**Figure 5:**
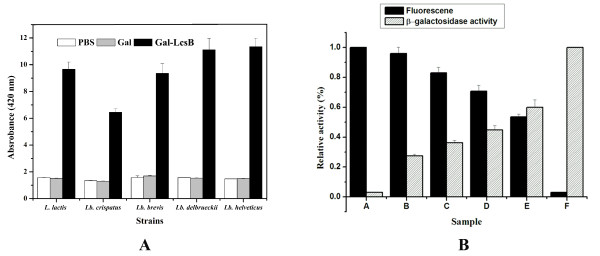
**Functional dispaly of beta-galactosidase (A) and two proteins simultaneously (B) on the LAB cells**. (A) PBS, Gal and Gal-LcsB were added to equal amounts of *L. lactis*, *Lb. crispatus*, *Lb. brevis*, *Lb. delbrueckii *and *Lb. helveticus*, respectively. After the binding experiment, the surface-exposed beta-galactosidase activities of various LAB cells were measured. (B) The samples which contained GFP-LcsB and Gal-LcsB fusion proteins at different ratio were added to the cells of *L. brevis *and fluorescence intenstiy and enzymatic activity were measured. Bar A, incubation with 1 volume of cell-free culture supernatant containing GFP-LcsB; bar B, incubation with 1 volume of cell-free culture supernatant containing 4/5 GFP-LcsB and 1/5 volume of Gal-LcsB; bar C, incubation with 1 volume of cell-free culture supernatant containing 3/5 GFP-LcsB and 2/5 volume of Gal-LcsB; bar D, incubation with 1 volume of cell-free culture supernatant containing 2/5 GFP-LcsB and 3/5 volume of Gal-LcsB; bar E, incubation with 1 volume of cell-free culture supernatant containing GFP-LcsB 1/5 and 4/5 volume of Gal-LcsB; bar F, incubation with 1 volume of cell-free culture supernatant containing Gal-LcsB. The mean value for three determinations was normalized to the activities for samples A and B, which were defined as 100%.

To further confirm the potential of the LcsB anchor, the beta-galactosidase gene (*gal*) from *Paenibacillus *sp. K1 [24] was fused to LcsB at its N-terminus instead of the GFP, and the Gal and Gal-LcsB proteins produced in *E. coli *Origami B (DE3) were also mixed with various LAB cells for 30 min to 60 min. As shown in Figure 5A, the beta-galactosidase activities on the surface of LAB cells tested were detected, indicating that the beta-galactosidase was anchored to the cell surface with the aid of LcsB and retained the beta-galactosidase activity. As a control, no galactosidase activity incubated with Gal or PBS was detected.

To test the possibility of displaying multiple proteins in a single cell, attempts were made to add the mixture of the GFP-LcsB and Gal-LcsB at different ratios to *Lb. brevis *cells. As a result, both fusion proteins, GFP-LcsB and Gal-LcsB could be detected on the cell surface of *Lb. brevis *using whole-cell fluorescence measurement and enzymatic activity assays, indicating that both proteins retained their function while presented on the cell surface (Figure 5B). The fluorescence intensity correlated well with the added GFP-LcsB content.

### Construction of the cell wall-targeting expression vector in *L. lactis *via LcsB anchor

**Figure 6:**
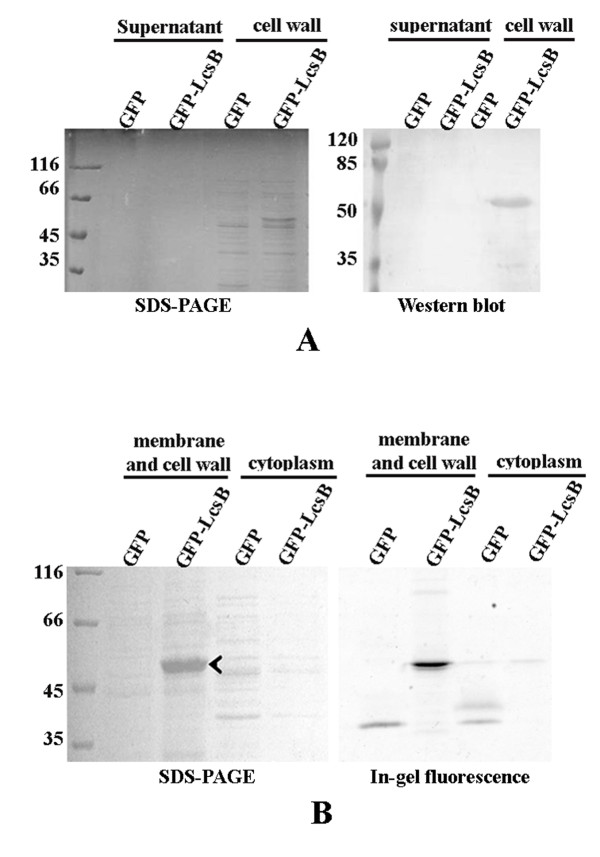
**Western blot analysis (A) and in-gel fluorescence assay (B) of *L. lactis *cell fractions**. (A) Western blot using the anti-GFP polyclonal antibodies were performed on fractionated protein extracts from *L. lactis *cells expressing GFP and GFP-LcsB. The positions of molecular mass standard are indicated. (B) In-gel fluorescence was monitored and the gel was stained with Coomassie (left). Intensities of in-gel fluorescent signals were plotted.

In this study, the results described above have proved that GFP and beta-galactosidase fused to LcsB region could be displayed on the surface of LAB cells. To further test whether the GFP or Gal fused to LcsB could be synthesized and efficiently exposed on the cell surface of *L. lactis *in vivo, the fused DNA fragment of *gfp:lcsB *or *gal:lcsB *was inserted into the expression vector pSec:Leiss:Nuc, which could secrete the nuclease into the medium under the nisin inducible promoter [25]. Expression of GFP and GFP-LcsB were confirmed by Western blot analysis using the GFP-specific polyclonal antibody. As shown in Figure 6A, the GFP-LcsB proteins were found in the cell wall fractions and no obvious band could be observed in the supernatant, suggesting that GFP was bound to the cell wall due to the activity of LcsB anchor. In-gel fluorescence assay was used to assess the integrity and activity of the GFP-LcsB fusion in the membrane and cell wall fractions. When the gel was exposed to ultraviolet light, an obvious fluorescence band with an approximate molecular weight of 50 kDa, representing the GFP-LcsB fusions, was clearly visible in the membrane and cell wall fractions (Figure 6B arrows). A small amount of GFP was also detected in the membrane and cell wall fractions by in-gel fluorescence. However, the fluorescence intensity of GFP-LcsB was markedly higher than those of GFP, indicating that GFP was transported to the membrane and the cell wall with the aid of the LcsB anchor and retained the fluorescence activity. The fluorescence bands of GFP and GFP-LcsB were also found in the cytoplasm fractions, suggesting that GFP and GFP-LcsB were successfuly expressed in *L. lactis*. Moreover, the cytoplasm of cells expressing GFP produced two fluorescence bands. We believed that the latter maybe the mature GFP without the signal peptide. In conclusion, all the results demonstrated that the GFP-LcsB fusion proteins were displayed on the cell wall of *L. lactis *and retained their fluorescence activity.

**Figure 7:**
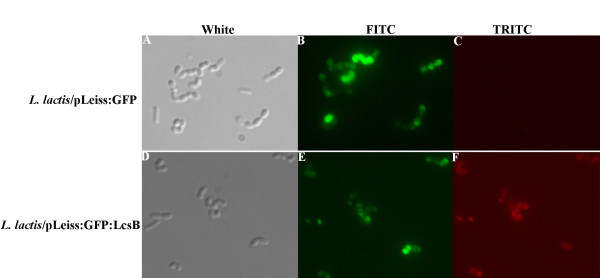
**Comparison of GFP expression on the surface of *L. lactis*/pLeiss:GFP and *L. lactis*/pLeiss:GFP:LcsB cells**. Panels A-F showed micrographs of the same field of *L. lactis*/pLeiss:GFP (A-C) or *L. lactis*/pLeiss:GFP:LcsB (D-F) after immunolabeling with antibodies using phase contrast (A and D), FITC (B and E), and TRITC (C and F) fluorescence microscopy.

Immunolabeling with specific antibodies is a useful tool to detect surface-exposed protein. The localization of the GFP on the external surface of *L. lactis *was determined by immunofluorescence microscopy. In Figure 7, panel A, B, C represented the *L. lactis*/pLeiss:GFP expressing GFP, and panels D, E, F represented the *L. lactis*/pLeiss:GFP:LcsB expressing GFP-LcsB fusions. The left, middle and right panels represented the same field of the sample viewed under light microscope, fluorescence microscope with FITC filter, and with rhodamine filter, respectively. As shown in Figure 7, the *L. lactis*/pLeiss:GFP:LcsB cells were positively decorated with red fluorescence, while no red fluorescence was observed on the cell surface of *L. lactis */pLeiss:GFP, suggesting that the GFP-LcsB were exposed to the cell surface of *L. lactis *with the aid of the LcsB anchor. Moreover, the red fluorescence was inconsistent with the intensity of green fluorescence, maybe because that the chromophore of GFP was influenced after the binding of antibody. Unfortunately, the expression of Gal-LcsB on the cell surface in *L. lactis *failed due to the improper enzyme folding in the cell wall or the low secretion efficiency of the Gal-LcsB (data not shown).

**Figure 8:**
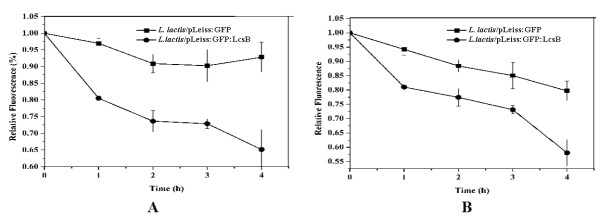
**SDS sensitivity assays for *L. lactis*/pLeiss:GFP and *L. lactis*/pLeiss:GFP:LcsB, respectively**. Results are presented as percentage of the fluorescence intensity of cells prior to the addition of 0.5% SDS (A) and 1% SDS (B). The data points represent the averages and standard deviations from four independent experiments.

Since externally added macromolecules can not penetrate the cell membrane, extracellular SDS treatment of intact cells has been used to provide evidence for the surface localization of target proteins. As shown in Figure 8, approximate 40% reduction of fluorescence intensity was observed when the *L. lactis*/pLeiss:GFP:LcsB was treated with 0.5% or 1% SDS for 4 hours, a relatively high reduction compared with the SDS pretreated *L. lactis*/pLeiss:GFP cells.

## Discussion

S-layer proteins form porous lattices composed of identical subunits which cover the whole cell surface and may constitute up to 20% of the total cell protein content. These properties make S-layer proteins an attractive anchor for the development of microbial cell-surface display systems [8]. In this study, we first developed a means for displaying heterologous proteins on the cell surface of LAB by using the cell wall binding domain of the S-layer protein SlpB as the cell wall anchor *in vitro *and *in vivo*.

To date, only a few S-layer protein genes of lactobacilli, such as *Lb. brevis*, *Lb. acidophilus*, *Lb. crispatus *and *Lb. helveticus *have been sequenced [2]. The C-terminal domain of *Lb. crispatus *JCM 5810 CbsA and *Lb. acidophilus *S_A _have recently been investigated with the capacity of binding the S-layer subunits to the teichoic acids of several bacterial species [3,4]. In this study, the C-terminal region LcsB of the S-layer protein SlpB from *Lb. crispatus *showed a high similarity with that of CbsA and S_A_, suggesting the LcsB could be used as the cell wall anchor to locate the heterologous proteins on the cell surface. We have proved that the heterologous proteins (GFP or Gal) were bound to the surface of various LAB cells *in vitro *and *in vivo*, opening up the possibilities for surface display of heterologous proteins (antigens or functional enzymes) on the LAB cells with the aid of LcsB anchor (Figure 2 and 5). Unlike the thus far reported the live *Lb. brevis *S-layer epitope constructs, which surface-displayed the small epitope as the part of the S-layer protein in the original hosts [12], our study described a versatile application of lactobacilli S-layers as an anchor for the surface display of more bulky polypeptide proteins on non-genetically modified bacteria.

A high binding capacity of the anchor protein is desirable for an efficient surface display system. The *Lactobacillus *cells saturated with GFP-LcsB contained 10^7 ^molecules per cells, which showed a high displaying capacity as same as the cA or Ly5C mediated surface display system [14-16]. The high surface density of LcsB on non-genetically modified LAB cells was compared favorably with those of other surface display systems, which relied on the expression of proteins on the surface of bacterial cells as a part of the S-layer. For example, 5 × 10^5 ^small epitopes could be obtained to surround a single *Lb. brevis *cell as part of S-layer [12]. Finally, LcsB from the S-layer protein of intestinal *Lb. crispatus *had a high affinity to *Lactobacillus *cells, providing the advantage of using various probiotic lactobacilli isolated from different environments as the host to present the functional proteins on the cell surface.

In addition to peptidoglycan, the rigid cell wall of lactobacilli is composed of secondary cell wall polymers (SCWP) such as teichoic acids, lipoteichoic acids, lipoglycans or teichuronic acids and so on [26]. Many gram-positive bacteria possess at the N-terminal part of their S-layer proteins the so called SLH motifs which are responsible for anchoring the S-layer proteins to SCWP [6]. No SLH motif has been found in the *Lactobacillus *S-layer proteins, and the neutral polysaccharide or (lipo)teichoic acids have been shown to be the cell wall ligand of *Lactobacillus *S-layer protein [3-5,7]. These features may explain why the GFP-LcsB bound to the entire surface of LAB cells (Figure 2). For *Lb. delbrueckii*, TCA-pretreated approach which mainly removed the cell wall-associated molecules (e.g., teichoic acids and polysaccharides) reduced the fluorescence intensity. For *Lb. crispatus *and *Lb. brevis*, TCA may get rid of the polysaccharides that hindered the binding of GFP-LcsB, leading to an increase in fluorescence intensity. Moreover, SDS treatment could remove the S-layer proteins of *Lb. crispatus *and expose the cell wall ligand of GFP-LcsB, resulting in the increase of displaying capacity. In addition, because of the remarkable deficiency of wall teichoic acids (WTA) the GFP-LcsB could not bind to the cell surface of *Lb. casei *[26], and the diversity of the (lipo)teichoic acids in LAB strains led to the different binding efficiency of GFP-LcsB.

For the present study, strain *L. lactis *N9000, which did not carry an S-layer protein, was chosen here as the model host because of its amenability to genetic engineering, the availability of a wide variety of genetic tools for this bacterium. The NIsin-Controlled gene Expression (NICE), which is one of the most widely used tools in regulating gene expression in *L. lactis*, was used to construct the cell wall-targeting expression system [27]. In this study, the expression vector pLeiss:GFP:LcsB, which could produce and target the GFP fusion protein to the cell wall in *L. lactis *N9000, was established. As expected, the *L. lactis*/pLeiss:GFP:LcsB was positively stained when incubated with GFP antibody and rhodamine labeled secondary antibody, resulting in a red fluorescence on the cells of *L. lactis*. Moreover, the red fluorescence was not as strong as green fluorescence and the reduction of fluorescence intensity of *L. lactis*/pLeiss:GFP:LcsB cells was low, suggesting that only a small part of GFP-LcsB was exposed to the surface of *L. lactis *cells. However, in-gel fluorescence assay of membrane and cell wall fractions showed that more GFP-LcsB fusion proteins were existent in the membrane as well as in the cell wall. We deduced that the GFP-LcsB could be poorly translocated across the cell wall and accumulated in the cell membrane. The protein targeting may be limited by the activity of the surface-located housekeeping protease HtrA [28] and the low-affinity interaction between the LcsB with the cell wall of *L. lactis*. To further improve the binding capacity, the *htrA*-deficient *L. lactis *or the colonizing commensal lactobacilli thus represent possible solutions.

## Conclusion

Sequence comparison revealed that the C-terminal region of S-layer protein SlpB of *L. crispatus *K2-4-3 (LcsB) had a high similarity with the cell wall-targeting domain of S_A _and CbsA, suggesting the LcsB could be sufficient to anchor the foreign proteins on the cell surface. Thus, the aim of this study was to test whether LcsB could be used to construct the surface display system as the cell wall anchor for LAB. First, a surface display system *in vitro *was establised and heterologous proteins (GFP and Gal) were successfully exposed to the surface of various LAB cells. Surprisingly, two protiens could bind to a single cell at a controlled ratio simultaneously, making the LcsB anchor attractive in displaying different epitopes on the cell surface. Moreover, the high loading capacity approaching 10^7 ^molecules per cell was advantageous in many applications. Finally, a versatile cell wall-targeting expression vector pLeiss:GFP:LcsB was constructed and successfully synthesized and exposed the GFP-LcsB fusion on the cell surface of *L. lactis*.

## Materials and methods

### Bacterial strains and culture conditions

All of the strains and plasmids used in this study are described in Table 1. The chicken intestine was chosen to isolate *Lactobacillus *strains which possessed the S-layer protein. All *Lactobacillus *strains were propagated in MRS broth (Oxoid, United Kingdom) at 37°C without aeration. *L. lactis *NZ9000 and *S. thermophilus *were grown in M17 broth (Oxoid, United Kingdom) supplemented with 0.5% (wt/vol) glucose (GM17) at 30°C or 42°C anaerobically, respectively. The *E. coli *strains were grown aerobically in Luria-Bertani (LB) broth at 37°C. To facilitate the selection of transformants, ampicillin, kanamycin, and tetracycline were used at 100 μg/mL, 34 μg/mL and 12.5 μg/mL for *E. coli*, respectively. Chloramphenicol was used at 10 μg/mL and 5 μg/mL for *E. coli *and *L. lactis*, respectively.

**Table 1:** Strains, plasmids used in this study.

Strain	Decription	Source
*E. coli *DH5α	supE44ΔlacU169φ80lacZΔM15hsdR17recA1endA1gyrA96thi-1relA1	Novagen
*E. coli *Origami B (DE3)	F-ompT hsdSB (rB- mB-) gal dcm lacY1 ahpC gor522::Tn10 (TcR) trxB::Kan (DE3)	Novagen
*Lb. crispatus *K2-4-3	Isolated from chicken intestine	Our lab
*Lb. johnsonii *K23	Isolated from chicken intestine	Our lab
*Lb. salivarius*	Isolated from chicken intestine	Our lab
*L. lactis *NZ9000	MG1363 pepN::nisRK. Most commonly used host of the NICE system	[27]
*Lb. brevis *K31	Wild type	Our lab
*Lb. casei *BL23	Kindly gifted by Hazebrouck	[36]
*S. thermophilus *K11	Wild type	Our lab
*Lb. helveticus *K17	Wild type	Our lab
*Lb. delbrueckii *subsp. bulgaricus ATCC 11842	Kindly gifted by M. van de Guchte	[37]
**Plasmid**		
pSec:Leiss:Nuc	pGK:Cmr; mature Nuc fused at 5' end with sequence of propeptide LEISSTCDA under the control of PnisA	[25]
pLeiss:GFP	pSec:Leiss:Nuc(NsiI/EcoRI::gfp)	This study
pLeiss:GFP:LcsB	pSec:Leiss:Nuc(NsiI/EcoRI::gfp-lcsB)	This study
pET-gfp	Ap^r^,pET-22b(+)(NdeI/HindIII::gfp-Taghis6)	[15]
pET-gfp-lcsB	Ap^r^,pET-22b(+)(NdeI/XhoI::gfp-lcsB-Taghis6)	This study
pET-gal	Ap^r^,pET-22b(+)(NdeI/HindIII::gal-Taghis6)	[15]
pET-gal-lcsB	Ap^r^,pET-22b(+)(NdeI/XhoI::gal-lcsB-Taghis6)	This study

### Extraction of S-layer proteins from *Lactobacillus *cells and cloning of the S-layer gene

Extracellular proteins were extracted from *Lactobacillus *cells on the method as described by Hagen et al [29]. Briefly, the cells of overnight culturs were harvested by centrifugation and washed twice with phosphate-buffer saline (PBS; 137 mM NaCl, 2.7 mM KCl, 10 mM Na_2_HPO_4_, 2 mM KH_2_PO_4_, pH 7.4), then treated with extraction buffer (2% SDS, 1% beta-mercaptoethanol) at 70°C for 10 min. The cells were pelleted by centrifugation at 3500 g for 5 min, and the supernatant (the cell surface-associated proteins) was analyzed by 12% SDS-PAGE.

The oligonucleotide primers used in this study are listed in Table 2. Standard recombinant DNA techniques were performd essentially as methods described previously [30]. Electroporation of *L. lactis *was performed with a Gene Pulser (2500 V, 200 Ω, 25 μF, 5 ms, Bio-Rad Laboratories) as previously described [31]. PCR products were generated using rTaq polymerase (TaKaRa, Japan).

**Table 2:** Primers used in this study

Primers	Sequences (5'-3')	Source
CbsA2F	GTACCAAGCCAAAGCAAGAC	[30]
CbsA2R	GTTTGAAGCCTTTACGTAAGTC	[29]
SF	CAAGTTCATCAACGCTGACAACATCG	This study
SR	GTAGTATGCATTGTGCATAATAGTCT	This study
SlpF	ACATTTTTTATATTTCAAGGAGGA	This study
SlpR	CGTTTTCCTTATATACTACTTAGACT	This study
8F	AGAGTTTGATCCTGGCTCAG	[35]
1541R	AAGGAGGTGATCCAGCCGCA	[35]
LcsB-HindIII	GCT**AAGCTT**AATTGGTTCGTTGCAC	This study
LcsB-XhoI	GTG**CTCGAG**GTTTGAAGCCTTTACGT	This study
GFP-NisI	GAG**ATGCAT**ATATGAGCAAAGGAGAAG	This study
Gal-NsiI	CGC**ATGCAT**TGATTAGCAGCAAATTACC	[38]
LcsB-EcoRI	GCG**GAATTC**TTAAAAGTTTGAAGCCTTTACGT	This study
GFP-EcoRI	CGC**GAATTC**TTAGTAGAGCTCATC	This study
Cm	ATGGACTTCATTTACTGGGTTTA	This study

Restriction enzyme cut sites are in bold.

Genomic DNA was extracted from *Lactobacillus *using the protocol of Walter et al [32]. The primers CbsA2F and CbsA2R designed to anneal to two conserved sequences localized between nucleotide 931-940 and 1296-1317 on the gene encoding the S-layer protein of *Lb. crispatus *[33], were used to amplify the region encoding the conserved part of the S-layer protein. To amplify the upstream or downstream region of the S-layer protein encoding gene, the ligation-anchored PCR was performed as described previously, with some modifications [34]. The chromosomal DNA of lactobacilli was digested with *Hae*III, and then the appropriate DNA fragments (approx. 2000 bp) were excised from the agar gel and purfied by Gel Extraction Kit (Omega, Doraville, USA) and ligated into the *Hae*III digested plasmid pSec:Leiss:Nuc. The ligated sample was purified with Cycle-Pure Kit (Omega, USA) and used to the next PCR amplification as the template. A set of the S-layer protein gene-specific primers (SF-SR) and the vector-specific primer Cm were used to amplify the upstream and downstream region of the S-layer protein gene, respectively. The fragments obtained were purified and sequenced. At last, the whole S-layer protein gene (*slpB*) was PCR amplified by primers SlpF and SlpR and cloned into the pMD18-T vector, resulting in the vector pMD18-S.

The 16S rRNA genes of the lactobacilli isolates were amplified by PCR using the universal primers 8F and 1541R [35] and directly sequenced by Huada genomic sequence corporation.

### Plasmids and DNA manipulations

The LcsB fragments were PCR amplified from the plasmid pMD18-S vector using the oligonucleotides LcsB-HindIII and LcsB-XhoI. The PCR products were digested with *Hin*dIII and *Xho*I and then inserted into the *Hin*dIII/*Xho*I sites of the plasmid pET-gfp and pET-gal, yielding plasmids pET-gfp-lcsB and pET-gal-lcsB, respectively. To construct the recombinant plasmids for protein expressing of GFP-LcsB and Gal-LcsB in *L. lactis*, PCR amplifications of *gfp-lcsB *and *gal-lcsB *were performed using the primers GFP-NsiI/LcsB-EcoRI and Gal-NsiI/LcsB-EcoRI, respectively. Subsequently, the PCR products were digested with *Nsi*I and *Eco*RI and inserted into the corresponding sites of pSec:Leiss:Nuc, resulting in plasmids pLeiss:GFP:LcsB and pLeiss:Gal:LcsB. As a negative control, the *gfp *fragments were amplified using the primers GFP-NsiI and GFP-EcoRI from the plasmid pET-gfp and digested with *Nsi*I/*Eco*RI, then the digested PCR products were ligated into the *Nsi*I/*Eco*RI digested plasmid pSec:Leiss:Nuc, generating the plasmid pLeiss:GFP. Transfromation of *L. lactis *by electroporation was performed as described previously, resulting in recombinants *L. lactis*/pLeiss:GFP, *L. lactis*/pLeiss:GFP:LcsB, and *L. lactis*/pLeiss:Gal:LcsB.

### Protein expression in *E. coli *and *L. lactis*

Gene expression was carried out as described in the pET system Manual (Novagen, USA) by using *E. coli *strain Origami (DE3). *E. coli *strains harboring the recombinant plasmids constructed above were grown overnight at 37°C in LB medium supplemented with 100 μg/mL ampicillin and 12.5 μg/mL kanamycin. Subsequently, the overnight cultures were 100-fold diluted in 500 mL of fresh LB broth and induced for the proteins expression with 1 mM isopropyl- beta-D-thiogalactopyranoside (IPTG) when the cultures reached an OD_600 _of 0.8. After further incubation for 4 hours at 30°C, cells were harvested, washed, and resuspended in 100 mL of PBS. Then, the cells were disrupted by sonication on ice at 400 W for two cycles (one cycle consists of 50 times of sonication for 3 s, intermission for 8 s), and the clear lysates were centrifuged at 12000 *g *for 30 min and filtered (0.22 μm; Millipore) to remove the cell debris. Subsequently, the clear lysates were applied to HisTrap™ FF crude columns (GE Healthcare, USA) equilibrated with the binding buffer (20 mM sodium phosphate, 500 mM NaCl, 25 mM imidazole, pH 7.4), then the columns were washed with washing buffer (20 mM sodium phosphate, 500 mM NaCl, 50 mM imidazole, pH 7.4) and the His-tagged protein was eluted in elution buffer (20 mM sodium phosphate, 500 mM NaCl, 500 mM imidazole, pH 7.4). Concentrations of purified proteins were determined using the Bradford protein assay. SDS-PAGE was used to analyze protein expression.

For the expression of heterologous protein in *L. lactis *under the control of *nis*A promoter, the recombinant *L. lactis *strains were grown in GM17 medium at 30°C overnight. 2% (W/V) of overnight cultures were inoculated in 5 mL of the fresh GM17 medium containing chloramphenicol (5 μg/mL), and incubated until the OD_600 _reached 0.4. Then, nisin (Sigma, USA) was added to cultures at a final concentration of 10 ng/mL. Growth was continued for 6 hours at 18°C to induce the protein experssion.

### Binding of LcsB fusion proteins to LAB

LAB strains were grown as mentioned above, and cells of 1 mL stationary-phase cultures were collected, washed twice and resuspended in 100 μL of PBS. In typical binding experiment, the cells in 100 μL of PBS were incubated for 30 to 60 min at 37°C with 1 mL of the clear lysates containing excessive purified GFP, GFP-LcsB, Gal and Gal-LcsB proteins, respectively. After binding, cells were collected by centrifugation at 10000 *g *for 5 min and resuspended in 1 mL PBS with a vortex mixing. After washed five times with PBS, cells were resuspended in 1 mL of PBS. Subsequently, cells bound with LcsB fusion proteins were analyzed by the whole-cell fluorescence measurement, fluorescence microscopy and beta-galactosidase activity.

LAB cells were harvested in the stationary phase, resuspended in PBS and adjusted to an OD_600 _of 1.0 before treatment with 0.2 volume of different chemicals. The chemical treatments were performed as follows: heating at 100°C for 10 min, 5% TCA at 100°C for 10 min, 10% SDS at 100°C for 10 min, and 90% acetone at room temperature for 10 min, respectively. After treatments, the cells were washed completely with PBS to remove any residual chemicals. The pretreated cells were subsequently used for LcsB binding assay as described above.

The cell-associated fluorescence was measured on LS-50B spectrofluorometer (Perkin-Elmer, USA) by excitation at 488 nm and emission at 511 nm with untreated and black 96-well polystyrene test plate. The background fluorescence of cells was subtracted to obtain the relative fluorescence units (RFU). The cell density was measured at 600 nm, and the whole-cell fluorescence per OD_600 _was calculated. The fluorescent cells were observed by fluorescence microscopy (Nikon Eclipse TE2000-S, Japan). The beta-galactosidase activity of the cells was assayed using o-nitrophenyl-b-D-galactopyranoside (ONPG) as a chromogenic substrate [24].

### Cell fractionation and in-gel fluorescence assay

The cells of *L. lactis*/pLeiss:GFP and *L. lactis*/pLeiss:GFP:LcsB induced by nisin were centrifuged at 5000 *g *at 4°C for 5 min, then the supernatant fraction was obtained according to the TCA/acetone method. Briefly, 100 μL of 100% TCA was mixed with 1 mL supernatant for protein precipitation. Subsequently, the mixtures were vortexed for 15 s and placed on ice for a minimum of 15 min. The pellets of protein were obtained by centrifugation at 14000 *g *for 10 min and washed twice with 100 μL acetone. After dried in air, the pellets were dissolved in 50 μL of 1× SDS loading buffer.

The cell-wall fraction was extracted according to the S-layer extraction method described above and the method of membrane and cell wall fractions extraction, respectively. Membrane and cell wall fractions were isolated from 10 mL cultures of *L. lactis *induced by nisin at 18°C for 6 hours using the following method. The cultures were harvested and resuspended in 10 mL PBS buffer. After disrupting the cells by sonication on ice at 400 W for three cycles (one cycle consists of 50 times of sonication for 3 s, intermission for 8 s), the resuspension was centrifugated for 10 min at 10000 *g *to remove the cell debris. The resulting supernatants were centrifuged for 30 min at 100000 *g *and the membrane and cell wall fractions were recovered in the pellet which were resuspended in 50 μL PBS and mixed with 1× SDS-PAGE nonreducing buffer (200 mM Tris-HCl (pH 8.8), 20% glycerol, 5 mM EDTA (pH 8.0), 0.02% bromphenol bule, 4% SDS, 0.05 M dithiothreitol). The sample was incubated at 37°C for 5 min before electrophoresis in standard 12% SDS-PAGE. Subsequently, the gel was rinsed with distilled water and exposed to a STORM840 scanner (Amersham, USA) to detect the fluorescence band.

### Western blot

The proteins on the SDS-PAGE gel were electrotransferred onto a polyvinylidene fluoride membrane at 200 mA for 2 hours. The membrane was blocked with 5% non-fat milk for 1 hour and then incubated overnight with the primary anti-GFP polyclonal antibody (Clontech, USA) at a dilution of 1:2000. The membrane was washed with TBST buffer (20 mM Tris-HCl (pH 7.4), 500 mM NaCl, 0.01% Tween 20) three times for 15 min each time and incubated with horseradish-peroxidase (HRP)-coupled secondary antibody (Solarbio, China) at a dilution of 1:300 for 1 hour. The bands were visualized with HRP-DAB kit (Tiangen, China) according to the instruction manual.

### SDS sensitivity assay and immunofluorescence analysis

The cells of *L. lactis*/pLeiss:GFP and *L. lactis*/pLeiss:GFP:LcsB induced by nisin for 6 hours were harvested and washed three times with PBS, and then adjusted to an OD_600 _of 1.0. SDS was added to each cell suspension to a final concentration of 0.5% and 1% (wt/vol). The fluorescence intensity of SDS-treated and SDS-nontreated cells was measured at hourly interval. Then, the ratio between the fluorescence intensity of SDS-treated and SDS-nontreated cells was measured at hourly interval.

The cells of *L. lactis*/pLeiss:GFP and *L. lactis*/pLeiss:GFP:LcsB induced by nisin for 6 hours were harvested and washed three times with PBS. Firstly, blocking was performed by additon of 3% BSA and incubated at room temperature for 30 min. Cells were collected and resuspended in PBS buffer containing 3% BSA and primary anti-GFP polyclonal antibody (diluted 1:100) for 1 hour. After washed three times with PBS, cells were incubated with the secondary rhodamine-conjugated goat anti-mouse IgG antibody (diluted 1:75) at room temprature for 1 hour in darkness. After washed three times and suspended with PBS, cells were observed by a Nikon fluorescence microscope equipped with xenon lamp and filter sets for FITC (485 nm for excitation and 505 nm for emission) and Rhodamine (540 nm for excitation and 600 nm for emission).

## Competing interests

The authors declare that they have no competing interests.

## Authors' contributions

JK defined the strategy and supervised the project. SH performed most of the experiments, made most of the date evaluation and interpretation, drafted parts of the manuscript. ZS participated in the identification of *Lactobacillus *and provided the character of *Lb. crispatus *K2-4-3. LH performed the Western blot and in-gel fluorescenc. WK helped to isolate *Lactobacillus *strains and PY participated in the binding experiment. All authors read and approved the final manuscipt.

## Author information

^1^State Key Laboratory of Microbial Technology, Shandong University, Jinan 250100, P. R. China and ^2^Scientific Research Center, Tsingtao Brewery Co.LTD, Qingdao, P. R. China.

## References

[B1] Sára M Sleytr UB S-layer proteins J Bacteriol 2000 182 859 868 10.1128/JB.182.4.859-868.2000 10648507 PMC94357

[B2] Åvall-Jääskeläinen S Palva A *Lactobacillus *surface layers and their applications FEMS Microbiol 2005 9 511 529 10.1016/j.femsre.2005.04.003 15935509

[B3] Smit E Jager D Martinez B Tielen FJ Pouwels PH Structural and functional analysis of the S-layer protein crystallisation domain of *Lactobacillus acidophilus *ATCC 4356: evidence for protein-protein interaction of two subdomains J Mol Biol 2002 324 953 964 10.1016/S0022-2836(02)01135-X 12470951

[B4] Antikainen J Anton L Sillanpää J Korhonen TK Domains in the S-layer protein CbsA of *Lactobacillus crispatus *involved in adherence to collagens, laminin and lipoteichoic acids and in self-assembly Mol Microbiol 2002 46 381 394 10.1046/j.1365-2958.2002.03180.x 12406216

[B5] Avall-Jääskeläinen S Hynönen U Ilk N Pum D Sleytr UB Palva A Identification and characterization of domains responsible for self-assembly and cell wall binding of the surface layer protein of *Lactobacillus brevis *ATCC 8287 BMC Microbiol 2008 8 165 181 10.1186/1471-2180-8-165 18828902 PMC2571106

[B6] Mesnage S Fontaine T Mignot T Delepierre M Mock M Fouet A Bacterial SLH domain proteins are non-covalently anchored to the cell surface via a conserved mechanism involving wall polysaccharide pyruvylation EMBO J 2000 19 4473 4484 10.1093/emboj/19.17.4473 10970841 PMC302060

[B7] Masuda K Kawata T Reassembly of a regularly arranged protein in the cell wall of *Lactobacillus buchneri *and its reattachment to cell walls: chemical modification studies Microbiol Immunol 1985 29 927 38 4079844

[B8] Leenhouts K Buist G Kok J Anchoring of proteins to lactic acid bacteria Antonie Van Leeuwenhoek 1999 76 367 376 10.1023/A:1002095802571 10532392

[B9] Marraffini LA Dedent AC Schneewind O Sortases and the art of anchoring proteins to the envelopes of gram-positive bacteria Microbiol Mol Biol Rev 2006 70 192 221 10.1128/MMBR.70.1.192-221.2006 16524923 PMC1393253

[B10] Narita J Okano K Kitao T Ishida S Sewaki T Sung MH Fukuda H Kondo A Display of alpha-amylase on the surface of *Lactobacillus casei *cells by use of the PgsA anchor protein, and production of lactic acid from starch Appl Environ Microbiol 2006 72 269 75 10.1128/AEM.72.1.269-275.2006 16391053 PMC1352207

[B11] Okano K Zhang Q Kimura S Narita J Tanaka T Fukuda H Kondo A System using tandem repeats of the cA peptidoglycan-binding domain from *Lactococcus lactis *for display of both N- and C-terminal fusions on cell surfaces of lactic acid bacteria Appl Environ Microbiol 2008 74 1117 1123 10.1128/AEM.02012-07 18156338 PMC2258587

[B12] Avall-Jääskeläinen S Kylä-Nikkilä K Kahala M Miikkulainen-Lahti T Palva A Surface display of foreign epitopes on the *Lactobacillus brevis *S-layer Appl Environ Microbiol 2002 68 5943 5951 10.1128/AEM.68.12.5943-5951.2002 12450814 PMC134443

[B13] Liu X Lagenaur LA Lee PP Xu Q Engineering of a human vaginal *Lactobacillus *strain for surface expression of two-domain CD4 molecules Appl Environ Microbiol 2008 74 4626 4635 10.1128/AEM.00104-08 18539799 PMC2504410

[B14] Bosma T Kanninga R Neef J Audouy SA van Roosmalen ML Steen A Buist G Kok J Kuipers OP Robillard G Leenhouts K Novel surface display system for proteins on non-genetically modified gram-positive bacteria Appl Environ Microbiol 2006 72 880 889 10.1128/AEM.72.1.880-889.2006 16391130 PMC1352190

[B15] Varma NR Yusoff K Ross E Foo HL Cell surface display system for *Lactococcus lactis*: a novel development for oral vaccine Appl Microbiol Biotechnol 2005 68 75 81 10.1007/s00253-004-1851-8 15635459

[B16] Hu S Kong J Kong W Guo T Ji M Characterization of a novel LysM domain from *Lactobacillus fermentum *bacteriophage endolysin and its use as an anchor to display heterologous proteins on the surfaces of lactic acid bacteria Appl Environ Microbiol 2010 76 2410 2418 10.1128/AEM.01752-09 20173067 PMC2849211

[B17] Völlenkle C Weigert S Ilk N Egelseer E Weber V Loth F Falkenhagen D Sleytr UB Sára M Construction of a functional S-layer fusion protein comprising an immunoglobulin G-binding domain for development of specific adsorbents for extracorporeal blood purification Appl Environ Microbiol 2004 70 1514 1521 10.1128/AEM.70.3.1514-1521.2004 15006773 PMC368406

[B18] Nomellini JF Duncan G Dorocicz IR Smit J S-layer-mediated display of the immunoglobulin G-binding domain of streptococcal protein G on the surface of *Caulobacter crescentus*: development of an immunoactive reagent Appl Environ Microbiol 2007 73 3245 3253 10.1128/AEM.02900-06 17384306 PMC1907123

[B19] Zarschler K Janesch B Kainz B Ristl R Messner P Schäffer C Cell surface display of chimeric glycoproteins via the S-layer of *Paenibacillus alvei* Carbohydr Res 2010 345 1422 31 10.1016/j.carres.2010.04.010 20513375 PMC4401010

[B20] Truppe M Howorka S Schroll G Lechleitner S Resch S Kuen B Lubitz W Biotechnological applications of recombinant S-layer proteins rSbsA and rSbsB from *B. stearothermophilus *PV72 FEMS Microbiology Reviews 1997 20 88 98

[B21] Sillanpää J Martínez B Antikainen J Toba T Kalkkinen N Tankka S Lounatmaa K Keränen J Höök M Westerlund-Wikström B Pouwels PH Korhonen TK Characterization of the collagen-binding S-layer protein CbsA of *Lactobacillus crispatus* J Bacteriol 2000 182 6440 6450 10.1128/JB.182.22.6440-6450.2000 11053389 PMC94791

[B22] Waldo GS Standish BM Berendzen J Terwilliger TC Rapid protein-folding assay using green fluorescent protein Nat Biotechnol 1999 17 691 695 10.1038/10904 10404163

[B23] Martínez B Sillanpää J E Smit E Korhonen TK Pouwels PH Expression of cbsA encoding the collagen-binding S-protein of *Lactobacillus crispatus *JCM5810 in *Lactobacillus casei *ATCC 393(T) J Bacteriol 2000 182 6857 6861 10.1128/JB.182.23.6857-6861.2000 11073938 PMC111436

[B24] Lu W Kong W Sun Z Kong J Ji M Cloning and expression of the beta-galactosidase gene of *Paenibacillus *sp. K1 in *E. coli* Journal of Shandong University (Natural Science) 2008 43 69 73

[B25] Ribeiro LA Azevedo V Le Loir Y Oliveira SC Dieye Y Piard JC Gruss A Langella P Production and targeting of the Brucella abortus antigen L7/L12 in *Lactococcus lactis*: a first step towards food-grade live vaccines against brucellosis Appl Environ Microbiol 2002 68 910 916 10.1128/AEM.68.2.910-916.2002 11823235 PMC126665

[B26] Kleerebezem M Hols P Bernard E Rolain T Zhou M Siezen RJ Bron PA The extracellular biology of the lactobacilli FEMS Microbiol Rev 2010 34 199 230 10.1111/j.1574-6976.2009.00208.x 20088967

[B27] Mierau I Kleerebezem M 10 years of the nisin-controlled gene expression system (NICE) in *Lactococcus lactis* Appl Microbiol Biotechnol 2005 68 705 717 10.1007/s00253-005-0107-6 16088349

[B28] Miyoshi A Poquet I Azevedo V Commissaire J Bermudez-Humaran L Domakova E Le Loir Y Oliveira SC Gruss A Langella P Controlled production of stable heterologous proteins in *Lactococcus lactis* Appl Environ Microbiol 2002 68 3141 3146 10.1128/AEM.68.6.3141-3146.2002 12039780 PMC123920

[B29] Hagen KE Guan LL Tannock GW Korver DR Allison GE Detection, characterization, and in vitro and in vivo expression of genes encoding S-proteins in *Lactobacillus gallinarum *strains isolated from chicken crops Appl Environ Microbiol 2005 71 6633 6643 10.1128/AEM.71.11.6633-6643.2005 16269691 PMC1287629

[B30] Sambrook J Fritsch EF Maniatis T Molecular cloning: alaboratory manual 1989 Cold Spring Harbor Laboratory Press

[B31] Holo H Nes IF High-Frequency Transformation, by Electroporation, of *Lactococcus lactis *subsp. *cremoris *Grown with Glycine in Osmotically Stabilized Media Appl Environ Microbiol 1989 55 3119 3123 16348073 10.1128/aem.55.12.3119-3123.1989 PMC203233

[B32] Walter J Tannock GW Tilsala-Timisjarvi A Rodtong S Loach DM Munro K Alatossava TL Detection and identification of gastrointestinal *Lactobacillus *species by using denaturing gradient gel electrophoresis and species-specific PCR primers Appl Environ Microbiol 2000 66 297 303 10.1128/AEM.66.1.297-303.2000 10618239 PMC91821

[B33] Horie M Kajikawa HS Toba T Identification of *Lactobacillus crispatus *by polymerase chain reaction targeting S-layer protein gene Lett Appl Microbiol 2002 35 57 61 10.1046/j.1472-765X.2002.01141.x 12081551

[B34] Sawa N Zendo T Kiyofuji J Fujita K Himeno K Nakayama J Sonomoto K Identification and characterization of lactocyclicin Q, a novel cyclic bacteriocin produced by *Lactococcus *sp. strain QU 12 Appl Environ Microbiol 2009 75 1552 1558 10.1128/AEM.02299-08 19139222 PMC2655440

[B35] Edwards U Rogall T Blöcker H Emde M Böttger EC Isolation and direct complete nucleotide determination of entire genes. Characterization of a gene coding for 16S ribosomal RNA Nucleic Acids Res 1989 17 7843 7853 10.1093/nar/17.19.7843 2798131 PMC334891

[B36] Hazebrouck S Pothelune L Azevedo V Corthier G Wal JM Langella P Efficient production and secretion of bovine beta-lactoglobulin by *Lactobacillus casei* Microb Cell Fact 2007 6 6 12 10.1186/1475-2859-6-6 17417967 PMC1853110

[B37] Van de Guchte M Penaud S Grimaldi C Barbe V Bryson K Nicolas P Robert C Oztas S Mangenot S Couloux A Loux V Dervyn R Bossy R Bolotin A Batto JM Walunas T Gibrat JF Bessières P Weissenbach J Ehrlich SD Maguin E The complete genome sequence of *Lactobacillus bulgaricus *reveals extensive and ongoing reductive evolution Proc Natl Acad Sci 2006 103 9274 9279 10.1073/pnas.0603024103 16754859 PMC1482600

[B38] Sun Z K W Kong J Expression of Lactase from *Paenibacillus *sp. K1 in *Lactococcus lactis* Journal of Shandong University (Natural Science) 2008 43 74 82

